# Mechanisms Underlying the Skin-Gut Cross Talk in the Development of IgE-Mediated Food Allergy

**DOI:** 10.3390/nu12123830

**Published:** 2020-12-15

**Authors:** Marloes van Splunter, Liu Liu, R.J. Joost van Neerven, Harry J. Wichers, Kasper A. Hettinga, Nicolette W. de Jong

**Affiliations:** 1 Internal Medicine, Allergology & Clinical Immunology, Erasmus Medical Centre, 3000 CA Rotterdam, The Netherlands; m.e.vansplunter@erasmusmc.nl (M.v.S.); l.liu@hagaziekenhuis.nl (L.L.); 2 Cell Biology and Immunology, Wageningen University & Research Centre, 6708 WD Wageningen, The Netherlands; joost.vanneerven@wur.nl; 3 FrieslandCampina, 3818 LE Amersfoort, The Netherlands; 4 Wageningen Food & Biobased Research, Wageningen University & Research Centre, 6708 WG Wageningen, The Netherlands; harry.wichers@wur.nl; 5 Food Quality & Design Group, Wageningen University & Research Centre, 6708 WG Wageningen, The Netherlands; kasper.hettinga@wur.nl

**Keywords:** skin-gut-axis, cutaneous sensitization, food allergy, atopic dermatitis, microbiota

## Abstract

Immune-globulin E (IgE)-mediated food allergy is characterized by a variety of clinical entities within the gastrointestinal tract, skin and lungs, and systemically as anaphylaxis. The default response to food antigens, which is antigen specific immune tolerance, requires exposure to the antigen and is already initiated during pregnancy. After birth, tolerance is mostly acquired in the gut after oral ingestion of dietary proteins, whilst exposure to these same proteins via the skin, especially when it is inflamed and has a disrupted barrier, can lead to allergic sensitization. The crosstalk between the skin and the gut, which is involved in the induction of food allergy, is still incompletely understood. In this review, we will focus on mechanisms underlying allergic sensitization (to food antigens) via the skin, leading to gastrointestinal inflammation, and the development of IgE-mediated food allergy. Better understanding of these processes will eventually help to develop new preventive and therapeutic strategies in children.

## 1. Introduction

Immune-globulin E (IgE)-mediated food allergy is characterized by a variety of clinical entities within the gastrointestinal tract, skin, and lungs, as well as systemically as anaphylaxis. IgE is the hallmark of allergic sensitization and, therefore, the most important antibody isotype in patients with atopic diseases. Sensitization is the process that leads to the presence of food-specific IgE in the serum and the skin, which predisposes to the development of food allergy. Several animal models have shown that epicutaneous allergen exposure, prior to oral challenge with the same antigen, induces allergic responses in the gastrointestinal tract [1,2,3,4]. Observations that children with atopic dermatitis (AD) and altered epidermal barrier function are more often sensitized to food antigens led to the hypothesis that exposure to a low dose of food antigen on the skin leads to sensitization, whereas early oral ingestion of food antigens (in a high dose) mediates tolerance. This hypothesis was first described by the group of Gideon Lack as the dual allergen hypothesis [5], and was recently updated and reviewed [6]. Several clinical trials in atopic children have shown evidence supporting the dual allergen hypothesis. AD is often the first manifestation of the atopic march and clear positive correlations have been seen between early-onset eczema (particularly for ages less than three months) and more severe eczema and the risk of developing food allergy [7]. Furthermore, AD can progress into asthma, a process mediated by thymic stromal lymphopoietin (TSLP) as demonstrated in mouse models of experimental asthma [8,9,10]. Next to this, AD is found to further increase the effect of exposure and sensitization to food allergens [11]. In the prospective LEAP (Learning Early About Allergy to Peanut trial) and LEAP-on studies, early introduction of peanut to 4–11-month-old children, with high risk of developing peanut allergy, significantly reduced the risk of developing peanut allergy by the age of 5 [12]. This high-risk population is identified as infants having AD and/or egg allergy, without having an established peanut allergy [13]. To interfere with the effect of AD, children with AD were extensively treated for their eczema with topical corticosteroids or emollients until remission in the PETIT study. The treatment of eczema was combined with either an early introduction of egg white or placebo (4–5 months of age) in a two-step protocol and resulted in a lower prevalence of egg allergy (9%) compared to children that were given placebo (38% egg allergic) at the age of 1 year [14]. This shows that optimal eczema treatment of AD patients, which resulted in lower SCORAD and POEM scores in both groups, is in itself not enough to prevent sensitization to food proteins. In contrast, early oral introduction of food does prevent sensitization to food proteins, as described in the dual allergen hypothesis [5]. So, optimal eczema treatment of AD patients can contribute in the prevention of sensitization; however, this only works in addition to oral introduction of food allergens.

How prior allergic sensitization via the skin progresses to food-induced anaphylaxis is not fully elucidated. Food-induced anaphylaxis is an immediate, adverse reaction, primarily triggered by cross-linking of allergen-specific IgE bound to the high-affinity IgE receptor (FcεRI) on mast cells (MCs) after re-exposure to the same food allergen [15]. However, only some individuals develop anaphylaxis, while others do not, independently of allergen-specific serum IgE levels. This suggests that other mechanisms than solely allergen-specific IgE are involved in the cascade of symptoms seen in food allergy. In this review, we will focus on the molecular and cellular mechanisms supporting the dual allergen exposure hypothesis and recent advances in understanding the interaction between immune responses in the skin and in the gut in the development of food allergy.

## 2. Skin Barrier and Skin Sensitization

The function of the skin epithelium is to provide a permeability barrier to maintain water and electrolytes homeostasis and an immune barrier, which facilitates commensal, but not pathogenic bacteria [16]. The skin is composed of epidermis and dermis. The epidermis is subdivided into the stratum corneum on the outside and inwards the stratum corneum is followed by the stratum granulosum, stratum spinosum, and stratum basale [17]. The stratum corneum is composed of keratinocytes differentiated into corneocytes and contains, among others: keratin filaments, filaggrin, and lipids [17]. Tight junctions are located in the stratum granulosum and are sealing the keratinocytes of the stratum corneum providing the permeability barrier [17]. Deeper into the skin through the epidermal barrier, the human dermis contains numerous immune cell types, such as Langerhans cells, mast cells, adaptive resident lymphocytes, and innate lymphoid cells (ILCs), together constituting the immune barrier.

Atopic dermatitis (AD) is a comorbid condition, which often precedes food allergy in patients. Furthermore, AD is a common inflammatory skin disease, which often develops during infancy and proceeds into adulthood. It has a relapsing character with pruritus eczematous flares. The pathophysiology of AD is multifactorial and includes genetic predisposition leading towards a defective skin barrier, dysregulated immune response, and microbial dysbiosis. Furthermore, environmental factors, such as allergens, micro-organisms, and toxins, influence the disease development [16,17]. Patients with AD have a significantly higher risk of developing food allergy [7]. This suggests that the skin is an important site of food allergen sensitization. In allergic sensitization via the skin, food antigens cross the disrupted epithelial barrier and mediate the release of danger signals and inflammatory cytokines TSLP and interleukin 33 (IL-33) through epithelial cells. These cytokines activate dendritic cells, which induce the differentiation of naive CD4+ T cells into a T helper cell 2 (Th2) phenotype. Clinical studies indicate that children who suffer from IgE-mediated food allergy are most likely sensitized through the gastro-intestinal tract and/or the skin in early infancy. Hill et al. has demonstrated in a multicenter large cohort study that early AD onset and severity are associated with high levels of IgE to food allergens, such as milk, egg, and peanut [18].

The most important genetic risk factors for AD are filaggrin (FLG) null mutations, which encodes for the epidermal protein FLG [19]. However, this FLG mutation alone is neither sufficient nor necessary to drive the development of AD. Patients with AD who carry the FLG mutation tend to have early onset, severe, and persistent skin disease and are more likely to be sensitized to multiple (food) allergens and to develop asthma [19]. Although a decreased barrier function is associated with increased intradermal allergen exposure, the mechanism by which this leads to allergic sensitization is not fully understood. Transepidermal water loss (TEWL) is a marker of epidermal dysfunction and highly correlated with altered epidermal lipid composition and structure in AD, independently of FLG mutation [20]. Increased TEWL at the age of 2 days was found to be correlated with AD and with being allergic to food later in life, at the age of one and two years, respectively. These results further emphasize the likelihood of the skin as an important site for sensitization at an early age [21,22].

An intact epithelial barrier which prevents the entry of antigens, pathogens, and irritants, and thereby the production of inflammatory cytokines, is important in the maintenance of homeostasis. The importance of an intact epithelial barrier is emphasized by the finding in human subjects that mutations in genes, encoding proteins that are involved in skin barrier integrity, such as FLG and SPINK5, are independent risk factors for peanut allergy [22,23,24,25]. Interestingly, the odds ratio for FLG mutations and peanut allergy is even stronger than for AD (5.3 vs. 3.1) [19]. Therefore, disrupted barrier function by FLG mutations alone or by AD in general leads to enhanced sensitization. However, allergen-specific IgE levels induced by sensitization do not correlate with the prevalence of food allergy and type 2 inflammatory reactions

Key messages:

- Skin is an important permeability and immune barrier.
- Disrupted skin barrier leads to increased sensitization to food allergens in the skin.

## 3. Environmental Factors Induce Sensitization to Food Allergens via the Skin

Other factors may also play an important role in the process of sensitization. Walker et al. have demonstrated that skin barrier mutations, together with exposure to environmental allergens, such as *Alternaria alternata* or house dust mite (HDM) extract, were required to drive the development of food allergen sensitization and anaphylaxis [26]. The exposure to environmental allergens was done after the skin of mice was wiped with 4% sodium dodecyl sulfate (SDS) as detergent, to resemble the use of cleaning wipes on infants, and this turned out to be essential for the absorbance of the topical applied environmental allergens. SDS is a key ingredient of soap, which can degrade corneodesmosomes and thereby reduce integrity of the stratum corneum resulting in type 2 immune responses [27]. Next to this, Cayrol et al. showed proteases from a whole range of allergens including *A. alternata* and HDM can process IL-33 full-length into a more biological active form of IL-33 inducing type responses [28]. In this paper, it was even suggested that the cleavage of full-length IL-33 by allergen proteases is used as an allergen sensing system. Exposure to environmental allergens and detergents may happen prior to the development of atopic dermatitis, as well as decreases the development of tolerance during oral consumption of the food allergen [26]. In patients with AD, epicutaneous application of HDM was shown to induce TSLP expression in both lesioned and unaffected skin [29].

The importance of oral tolerance to food allergens is emphasized by the study of Han et al., where the development of food allergy could be blocked when the allergen was ingested by mice prior to skin exposure [1]. Strid et al. found that epicutaneous exposure to peanut protein 20 days prior to ingestion of a tolerogenic dose of peanut protein completely abolished oral tolerance induction in mice, whereas epicutaneous exposure 6 days prior to ingestion only partly disrupted oral tolerance induction [30]. Even in already oral tolerant mice, epicutaneous exposure of peanut protein resulted in increased IL-4 levels and increased peanut-specific IgE levels, thus demonstrating an increase in sensitization to peanut [30].

In humans, Leung et al. showed through RNA sequencing that non-lesioned skin of 62 children with AD and food allergy had unique properties associated with an immature skin barrier and type 2 immune activation [31]. Patients with AD and food allergy exhibited a high dendritic cell activation in their non-lesioned skin, which is comparable to that of the lesioned skin of all AD participants. Furthermore, FLG was found to be downregulated in both lesioned and non-lesioned skin of patients with AD [31,32]. Taken together, a decreased skin barrier function (possibly induced by detergents and intrinsic genetic defects), in combination with exposure of the skin to food allergens with meals and dust containing HDM, *A alternata*, or *Staphylococcus aureus*, likely synergize to promote sensitization to food allergens and the subsequent development of food allergy.

Key messages:

- Detergents and environmental allergens, like house dust mite or *Alternaria alternata* allergens, can disrupt skin barrier.
- Cutaneous exposure of allergens prior to ingestion leads to increased sensitization.
- Tolerance is induced if allergens are ingested prior to cutaneous exposure.

## 4. TSLP-Mediated Type 2 Inflammation in the Skin

Thymic stromal lymphopoietin (TSLP) is an epithelial cytokine, expressed mainly by epithelial cells of the skin, lungs, and intestine [33,34]. TSLP, in mice, was shown to be induced by cutaneous exposure to food antigens and upon skin barrier disruption [2,3,35]. In a Korean birth cohort, skin epithelial expression of TSLP at two months of age has been linked to the development of AD at 24 months of age [36]. TSLP is found to regulate naive T cell differentiation towards an inflammatory phenotype by conditioning dendritic cell (DC) maturation as antigen presenting cells [37]. These TSLP-DCs induce a unique type of Th2 cells through the OX-40 ligand that produces the classical type 2 pro-inflammatory cytokines (IL-4, IL-5, and IL-13) together with tumor necrosis factor (TNF)-α and no production of IL-10 [34,38]. Furthermore, in lesioned human AD skin samples, it was shown that high TSLP production leads to activation and migration of Langerhans cells from the epidermis towards the dermis and an increase of activated DCs in the dermis [34].

In mice, TSLP-activated DCs express OX40L, as well, and it was shown that OX40L-OX40 interaction between DC and T cells induced IL-3 production by naive T cells, resulting both in recruitment of basophils in the skin-draining lymph nodes, as well as IL-4 expression of T cells [39]. In mice with an atopic dermatitis-like skin, cutaneous food allergen sensitization induces an expansion of TSLP-elucidated basophils in the skin, which is sufficient to promote the development of IgE-mast cell mediated food allergy after oral antigen exposure [3,4]. Moreover, clinical signs of food allergy are significantly reduced after epicutaneous sensitization in mice whose basophils cannot produce IL-4. In addition, IL-4 depletion in epicutaneous sensitized mice results in a diminished IgE-mediated anaphylaxis response upon an oral challenge in mice [40]. Taken together, this suggests a critical role for IL-4 derived from TSLP-induced basophils in the sensitization to food allergens in the skin, and the development of food allergy. The importance of TSLP is also noted in eosinophilic esophagitis, a food allergy-associated inflammatory disease, where skin-derived TSLP results in basophil-mediated disease activation in humans, which was IgE-independent (based on mice experiments) [2].

Key message:

- Disrupted skin barrier leads to increased sensitization to food allergens in the skin. This process is mediated by TSLP-induced DC and basophils, producing IL-4 and resulting in enhanced type 2 responses.

## 5. Major Role for Type 2 Innate Lymphoid Cells (ILC2) and Epithelial Cytokines in the Development of Food Allergy

In recent years, it has become clear that particularly ILC type 2 cells (ILC2s) play an important role in food allergy and these cells are considered as the innate counterparts of adaptive T helper 2 cells. Barrier epithelial cells, such as skin keratinocytes, lung cells, and intestinal epithelial cells, are found to be crucial in recruiting these immune cells by producing chemokines. AD-like disorders can even be induced by overexpression of the chemokine CCL17 by keratinocytes [41]. Furthermore, barrier cells can determine type 2 immunity by controlling the activation of DCs and ILC2s through the secretion of the epithelium-derived cytokines TSLP, IL-25, and IL-33 [41].

One of these barrier cells are the so-called tuft cells. Tuft cells (or brush cells) produce IL-25, a distinct IL-17 cytokine member (IL-17E), upon inflammation and these epithelial cells are located in the intestine and trachea [36,42]. Murine strains that lack the IL-25 receptor are found to be more resistant to developing IgE-mediated food allergy after oral intake [1,43]. IL-25 stimulation, together with CD4+Th2 cells, that are induced after allergic sensitization, cause ILC2s to produce large amounts of IL-5 and IL-13, resulting in the development of food allergy in mice [43]. Furthermore, IL-13 produced by ILC2s and/or Th2 cells can promote the differentiation and expansion of tuft cells, resulting in a positive feedback loop [42].

ILC2 can activate dendritic cells and promote a Th2 cell-mediated immune response, and expand in an antigen-independent manner in the presence of TSLP, IL-25, and IL-33 [41]. TSLP is hereby the most important factor for ILC2 survival, whereas IL-33 mainly results in ILC2 activation, although the combination of IL-25, IL-33, and TSLP results in the highest cytokine production [44]. As a result, ILC2s produce large quantities of Th2 cytokines, such as IL-5, IL-9, IL-13, and, to a lesser extent, IL-4, as reviewed by Reference [36,45]. In addition, in particular the cytokines IL-4 and IL-13, can disrupt allergen-specific regulatory T cell (Treg) induction and proliferation, resulting in fewer Tregs and a decrease in their suppressive functions [46]. In addition to this, allergen-specific Tregs were found to have a more Th2-skewed profile, with the production of IL-4 in both mice and human [46]. Furthermore, these ILC2-derived cytokines can enhance mucosal mast cell activation and ILC2s can be activated by mast cells in an IgE-dependent way, creating a positive feedback loop, thereby further promoting the induction of food allergies in mice [47,48].

IL-33 is another epithelial cytokine and is constitutively expressed in high levels in epithelial cells. IL-33 is released whenever cells are activated via adenosine triphosphate (ATP) or when cells are damaged or become necrotic [49]. Not only keratinocytes produce IL-33; fibroblasts, endothelial cells and epithelial cells produce IL-33, as well. Whether immune cells are bona fide producers of IL-33 is debated as often only IL-33 mRNA expression is reported [50]. During inflammation, as is the case in AD, IL-33 levels are elevated in skin lesions [51,52] and serum [53]. These serum IL-33 levels correlate with AD severity [36,53]. IL-33 and IL-4 can both downregulate FLG in keratinocytes and thereby further affect the skin barrier and possible entrance of allergens [54]. In an AD-like mouse model, it was shown that IL-33 could induce the atopic march and gastrointestinal allergy, independently from TSLP [55].

The IL-33 receptor IL33R/ST2 (suppression of tumorigenicity 2) is found to be increased in skin lesions of patients with AD [51]. Galand et al. found that IL-33 is released after mechanical skin injury in mice and induces IgE-mediated mast cell degranulation, although IL-33 had no direct effect on specific IgE levels in serum or on Th2 responses [56]. In humans, IL-33 mRNA expression is also increased after tape stripping of the skin [56]. Tape stripping of the skin is used as a model for scratching. Besides, IL-33 activates mast cell degranulation in humans in vitro [57]. In patients with AD, more ILC2 cells are found in skin biopsies from lesions compared to skin biopsies of healthy donors [52,58]. In a mouse model, ILC2s proved to be necessary for the development of an AD-like phenotype, even independently of the adaptive immune system [58]. In AD patients, ILC2 cells have a higher expression of receptors for IL-25, IL-33, and TSLP [52]. When stimulating skin-derived ILC2 cells from healthy donors ex vivo, only IL-33 or the combination IL-33, together with IL-25 and TSLP, induced type 2 cytokines IL-5 and IL-13, but no IL-4 [52]. Besides, IL-33R expression was upregulated after IL-33 stimulation and IL-33 was more potent than TSLP to induce migration of ILC2s.

Next to this, it was shown in an in vivo experimental model that HDM allergic patients have a higher infiltration of lymphocytes and (ST2-positive) ILC2 cells and a higher IL-4, IL-5, and IL-13 level in blister fluid upon intra-epidermal injection of HDM compared to healthy subjects [52]. Furthermore, it was confirmed in a mouse model that ST2-positive skin DCs drive the development of Th2 responses to peanut, resulting in peanut allergy upon epicutaneous peanut exposure [59]. So, IL-33 is involved in acute reactions to consumed food by acting directly on mast cells and enhancing IgE-mediated activation, as well as inducing ILC2 cells and activation of DCs that drive Th2 cell responses [52,56,59]. On the other hand, in mice, it is shown that IL-33 can induce epithelial tissue repair by activating the production of amphiregulin by ILC2s [60,61] or Tregs [62].

In short, activation of ILC2s by local epithelial cytokines IL-33 and TSLP has been shown to play a major role in the development of food allergy. While Th2 cells, by producing IL-4, IL-5, and IL-13, were initially believed to be the only major players driving the type 2 immune response, our current knowledge indicates that the type 2 immune response is mediated by the cooperative actions of Th2 cells and ILC2s and can be induced by scratching. Neutralizing these type 2 cells or their secreted cytokines via, e.g., monoclonal antibodies, used in anti-IL-4 or anti-IL-5 therapy can be a useful approach for patients with an already disrupted skin barrier who have an increased chance of developing food-induced allergy.

Key messages:

- Skin damage results in the release of IL-33, TSLP.
- Specifically, IL-33 cells activate DCs and ILC2 cells.
- Through activating ILC2 cells and DCs, epithelial cytokines, e.g., TSLP and IL-25, can mediate a type 2 inflammation reaction in an antigen independent manner.

## 6. How Can Pruritus Lead to Food-Induced Anaphylaxis?

Pruritus or itch is the unpleasant sensation that causes an urge to scratch [63]. Pruritus can have multiple causes, such as local nerve fiber compression or degeneration of nerve fiber in the peripheral or central nerve system or, in the case of dermatological pruritus, due to type 2 immune responses in the skin [63,64].

IL-33 is very important in the crosstalk between the skin and gut. In the study of Savinko et al., ‘scratching’ of the skin by tape stripping affected 10% of the mouse total body surface area, which resulted in a significant 2-fold increase in circulating levels of IL-33 [51]. In AD patients, around 20% of the total body surface area is affected, and the median IL-33 level in serum from patients with AD is more than 10-fold that of healthy control subjects [53,65]. Interestingly, in mice, this IL-33 increases the number of mucosal MCs in the small intestine via ILC2 activation [56,66]. Furthermore IL-33 enhances IgE-mediated degranulation of MCs in the gut, which leads to the development of an anaphylactic response to ingested food allergens. MCs and basophils are essential in anaphylactic responses by releasing mediators into the circulation [15]. However, not all sensitized individuals who have food allergen-specific IgE antibodies develop food allergy, and serum concentration is not a predictive marker for allergy severity [67]. Altogether, these results indicate that IL-33 released on mechanical skin injury as a replacement for scratching can potentially target ST2-expressing cells, including MCs at distant sites.

**Figure 1:**
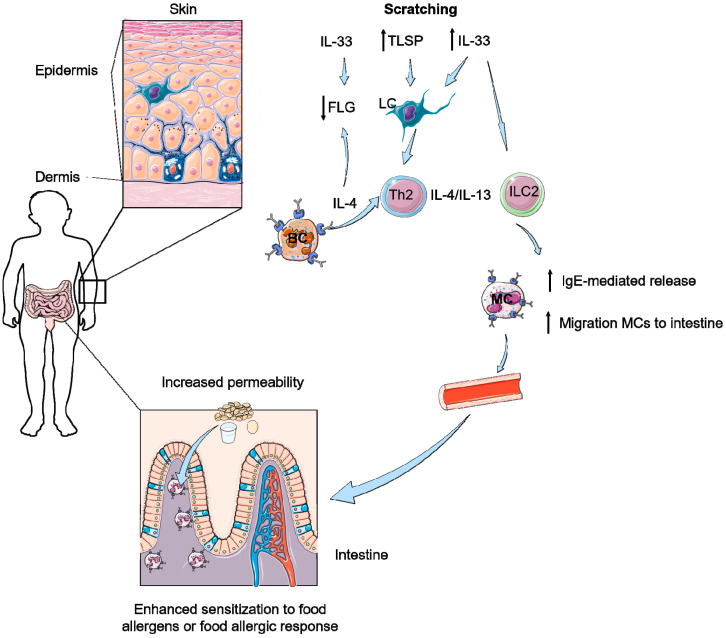
Scratching can further result in a decreased barrier function of the skin and the intestine. Scratching elicits thymic stromal lymphopoietin (TSLP) and IL-33 responses in skin activating Langerhans’ cells (LC), innate lymphoid type 2 cells (ILC2) and T helper 2 cells (Th2). Furthermore, IL-4 production of basophils (BC) enhances the type 2 responses and leads do a decrease of filaggrin (FLG) expression in combination with IL-33. Due to type 2 responses both IgE-mediated release of mast cells (MC) and migration of MCs to the intestine is increased. This results in an increased permeability of the intestine and therefore of an influx of food allergens, potentially leading to enhanced sensitization or allergic responses to these food antigens.

**Figure 2:**
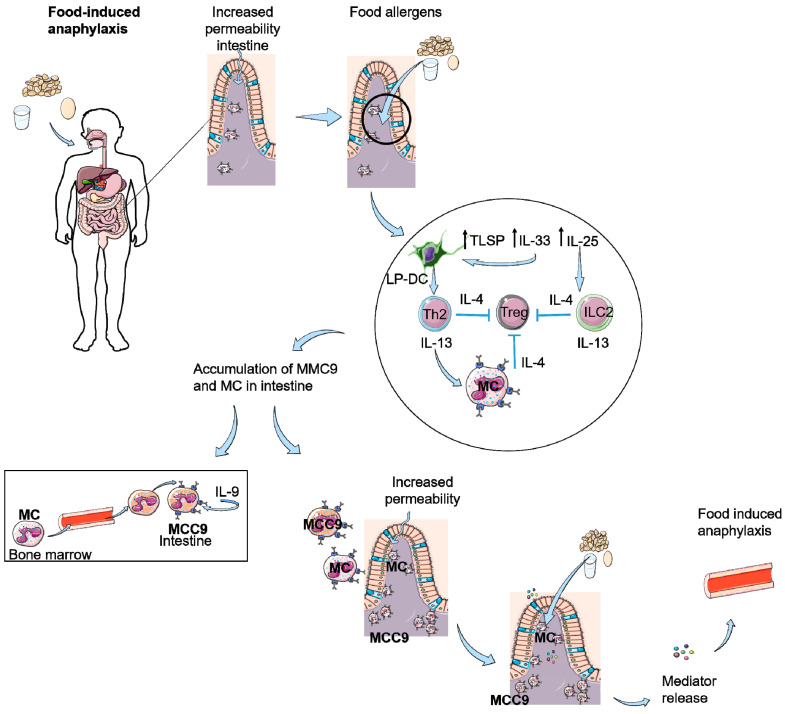
Food-induced anaphylaxis as a result of scratching or decreased skin barrier function. Intestinal permeability is increased due to influx of mast cells (MC) as a result of scratching or damaged skin. This results an increased entrance of food antigens in the intestine eliciting the production of TSLP, IL-33, and IL-25. IL-25 can activate ILC2 cells and IL-33 and TSLP activate dendritic cells in the lamina propia of the intestine (LP-DC), which activate Th2 cells. Th2 cells and ILC2 cells produce IL-4 and IL-13, resulting in inhibition of Tregs and stimulation of MCs. This leads to an accumulation of (sensitized) MC and IL-9 producing mucosal mast cells (MMC9) in the intestine, which causes an increased permeability of the intestine. Food allergens can passage the epithelial barrier, resulting in IgE-mediated degranulation of the MCs and as a result of mediator release, food-induced anaphylaxis.

Intestinal MC expansion is associated with susceptibility to food-induced anaphylaxis and increased intestinal MC load correlates with an increased severity of food-induced anaphylaxis [68]. Furthermore, tape stripped epicutaneously sensitized mice and not orally immunized mice show expansion of intestinal MCs and IgE-mediated anaphylaxis after a single oral antigen challenge [69]. Tape stripping in mice induces intestinal tuft cells to produce IL-25 at the same time. This IL-25 activates and expands ILC2s in skin and small intestine and mediates the release of IL-4 and IL-13, which in turn activates tuft cells to produce IL-25 in a positive feedback loop [42,66]. ILC2-produced IL-4 and IL-13 was essential, and not IL-5 and IL-9, to increase the intestinal MC load in this mouse model, which was all independent of T cells [66]. Intestinal MCs control intestinal permeability and, therefore, systemic absorption of food antigen and food anaphylaxis; see Figure 1 and Figure 2 [68,70]. Furthermore, it is known that sensitization towards food antigens can also occur throughout the gastro-intestinal tract [71]. Especially the increased intestinal permeability could lead to enhanced sensitization to food antigens in the intestinal tract, potentially followed by an allergic response or even an anaphylactic response to these food antigens. These effects of IL-25 and IL-33 in combination with TSLP-induced IL-4 production by basophils in the skin all result in enhanced IgE-mediated mast cell degranulation in the intestines; hence, IL-25, IL-33, and TSLP are key players in the skin-to-gut axis. That all three epithelial cytokines, IL-25, IL-33, and TSLP, play a role in the induction of food allergy was proven by Khodoun et al. In mice, they showed that only treatment with a cocktail of the three monoclonal antibodies against IL-25, IL-33, and TSLP, and not a single treatment, was sufficient to inhibit development of murine food allergy [72].

Additionally, the number of intestinal MCs was higher in duodenal biopsies of patients with AD compared to non-AD patients [66]. The authors conclude that increased intestinal MCs and permeability can be elicited by scratching, which play an important role in promoting food anaphylaxis in patients with AD. Therefore, interventions that inhibit scratching may be useful in dampening the severity of food allergy in these patients by decreasing their intestinal MC load [66].

A specific group of intestinal mucosal mast cells are found to be associated with IgE-mediated food allergy [73]. These cells have been identified as IL-9-producing mucosal MCs (MMC9s) and are mainly located in the lamina propria of the small intestine in mice [74]. So far, in human subjects, an increase in IL-9 producing cells are identified in duodenal biopsies of food allergic patients compared to healthy controls, based on qPCR [74]. Furthermore, in humans, expression levels of IL-9, IL-13, and MC-specific transcripts are associated with food allergic patients who develop comorbid allergic diseases, such as eczema and urticaria [74]. However, no flow cytometric analysis or immunohistochemistry analysis has been performed of these IL-9 producing cells, hence we do not know if these cells are real MMC9 cells. Therefore, all data further discussed in this section is based on murine models. MMC9s function as type-2-promoting innate myeloid cells by producing IL-9 and IL-13 cytokines in response to IL-33, but not IL-25, and secrete histamine and other MC mediators upon antigen-induced IgE-complex crosslinking [74]. In mucosal tissues, MC expansion is dependent on the Th2 and Th9 cytokines IL-3 and IL-9 [73]. Allergic symptoms are reduced in IL-9-deficient mice, whereas intestinal mastocytosis, intestinal permeability, and intravascular leakage are observed in mice overexpressing IL-9, leading to a predisposition to oral antigen sensitization [75]. Furthermore, cross-linking on the surface of an antigen-specific IgE/FcεR-complex promotes the proliferation of MMC9s and MCs [46,74]. Of note, MMC9s seem to derive from mast cell progenitor cells from the bone marrow and are able to mature into mucosal mast cells with a reduced IL-9 production [74]. Levels of both MMC9s and MCs are increased after repeated intragastric ovalbumin (OVA) challenges from a basal level of 0.5% to 9% of total mononuclear cells in the small intestine in sensitized mice, resulting in the development of experimentally induced food allergy [74]. In this experimental food allergy model, mice are sensitized twice with OVA (day 0 and day 12) and are intragastrically challenged six times with OVA between day 25 and day 36, resulting in a food allergic reaction towards OVA. In addition, Th2 cells are increasing at the same time from 0.5 to 4%, and Chen et al. show that IL-4 and Th2 cells are required for the induction of MMC9s resulting in experimentally induced food allergy [74,76]. However, ILC2 and basophil levels remained constant. Similar results were obtained in skin-sensitized mice. In addition, intestinal MCs can provide an IL-4 signal to induce regulatory T cell reprogramming toward a Th2-cell-like lineage, resulting in the impairment of regulatory T cell function and the loss of tolerance [46]. In addition to Th2 cell activation, naive T cells are shown to differentiate to Th9. Th9 cells also secrete IL-9 cytokines and further promote the accumulation of tissue residing mast cells in mice [77].

In summary, scratching affects the barrier function of the skin and leads to the release of IL-33 in the skin and increased IL-33 levels systemically; see Figure 1. A reduced barrier function of the skin due to FLG mutation or detergents, sometimes in combination with adjuvant activities of microbial ligands, ultimately result in the induction of IL-33 and TSLP. TSLP and/or IL- 33 released by keratinocytes synergize with IL-25 released by intestinal tuft cells to expand ILC2s and increase their expression/production of IL-4 and/or IL-13, as depicted in Figure 1 and Figure 2. ILC2-derived IL-4 and IL-13 target MCs to cause their expansion in the gastrointestinal mucosa, increase IgE-dependent degranulation of MCs and stimulate DCs to reduce allergen-specific Tregs. Furthermore, cutaneous sensitization induces TSLP activation of basophils that produce IL-4, production of type 2 cytokines IL-5 and IL-13, leading, as well, to an accumulation of mast cells in the intestine [3,30,40]. The accumulation of MCs in the gut results in a reduced gut barrier and hence a higher permeability of the gut barrier for food allergens, which leads to an enhanced risk for IgE-mediated degranulation of MCs and for the development of anaphylactic responses after exposure to food allergens. Taken together, these observations illustrate the existence of a skin-to-gut crosstalk in which mechanical skin injury can promote food-induced anaphylaxis by driving intestinal MC expansion, in addition to facilitating sensitization to food allergens.

Key messages:

- Scratching induces enhanced IL-33 levels in the skin and in serum.
- IL-33 together with IL-4 and Th2 cells are able to induce accumulation of mast cells and IL-9 producing mucosal mast cells (MMC9) in the intestine.
- IL-33 results in more IgE-mediated degranulation of these MCs and MMC9 cells, leading to food allergy.
- Scratching increases numbers of intestinal mast cells and increased permeability of the intestines resulting in the development of food allergy.
- A skin-to-gut axis is inevitable as food allergy symptoms in the intestine apparently can be induced by increased IL-33 levels in serum, which is induced by a damaged skin barrier due to scratching or AD.

## 7. The Role of Skin Microbiota in the Development of Food Allergy

In the development of AD, two hypotheses have been popular throughout the past decades. First, the ‘inside to outside-hypothesis’ was developed, in which the gut microbiota and the immune system were responsible for the decreased skin barrier function, leading to AD and allergy [78,79]. Later on, the ‘outside to inside-hypothesis’ became more dominant, in which skin barrier dysfunction was seen as a driver of AD development, which in turn leads to the activation of the immune system, resulting in a further reduced and affected skin barrier [80]. This review is mostly based on the ‘outside to inside-hypothesis’ and therefore we started from the skin barrier and its relation to allergy. The skin epithelial barrier is colonized with microbiota, and this microbiota diversifies throughout life [81]. Atopic dermatitis is in general associated with a lower diversity in the skin microbiota [82]. Staphylococcus aureus (*S. aureus*) colonization is highly associated with atopic dermatitis and eczema severity [83,84,85] and is found to cause a (partial) reduction of microbial diversity [82]. In the LEAP and LEAP-on studies, *S. aureus* colonization was found in children at 4 to 11 months of age (~22% on skin and ~18% in nose), which decreased to 8% on skin and 18% in nose at an age of 60 months [85]. In contrast, a recent birth cohort study revealed that in 1-year-old infants with AD, there was no dysbiosis in microbial communities and these infants’ microbiome were not (yet) colonized by *S aureus*. However, AD-affected children had less commensal Staphylococci compared to healthy children [81]. *S. aureus* is found to release δ-toxin, which triggers degranulation of mouse-derived mast cells in vitro and promotes both innate and adaptive type 2 responses in vivo in mice [86]. Pre-incubation of allergen-specific IgE on mouse-derived mast cells even resulted in a synergistic degranulation effect of *S. aureus* derived δ-toxin in the absence of antigen [86]. Exposure of mouse skin to Staphylococcus enterotoxin B (SEB), together with food allergens (soy, ovalbumin or peanut), can induce the Th2 phenotype via IL-33 stimulation of skin-draining DCs and induce food allergy [59]. Interestingly, not all food allergens needed an exogenous adjuvant (SEB or cholera toxin): cow’s milk allergen α-lactalbumin, green bean, and soy did need adjuvants to be able to induce sensitization, whereas cashew nut and peanut had intrinsic ‘adjuvant activities’ themselves [59]. Furthermore, only the combination of SEB and ovalbumin and not the single treatments resulted in Th2 responses in mice, and local mast cell activation and degranulation in the jejunum of these mice [87]. Next to SEB, other pathogenic factors, such as staphylococcal peptidoglycan or pertussis toxin, induced a Th2 polarization but not lipopolysaccharide (LPS) [87]. In line with these results, infiltrating T cells specific for SEB have been found in skin of AD patients [84]. These results underline that exposure of food allergens on skin, sometimes in combination with adjuvants in the form of microbial ligands or non-microbial ligands (e.g., detergents), is needed to develop food allergy.

A possible role for *S. aureus* colonization in the development of food allergy has also been proposed in human studies. An increase in the relative abundance of *S. aureus* in non-lesioned skin of AD patients with and without a food allergy compared to non-atopic controls was found in the study Leung et al. Next to this, a trend was observed of increased relative abundance of *S. aureus* in lesioned skin of AD patients with a food allergy compared to AD patients without a food allergy [31]. In the LEAP and LEAP-on studies, *S. aureus* colonization was related to more persistent egg white allergy and higher chances of having a peanut allergy at 60 months of age [85]. Furthermore, higher levels of specific-IgE levels to egg white, cow’s milk, and peanut were found [85]. Interestingly, these associations were independent of eczema severity.

Key messages:

- *Staphylococcus aureus* colonization is related to reduce microbial diversity in the skin and increased prevalence of atopic dermatitis and food allergy.

## 8. The Role of Intestinal Microbiota on the Development of Atopy and Atopic Dermatitis

Next to the skin-to-gut axis, there has also recently been more interest in the role of the gut microbiota in skin diseases, such as acne, psoriasis, and atopic dermatitis [88,89,90]. Another link between the skin and gut is the use of epicutaneous immunotherapy in the treatment of food allergy, where patches with food allergens are placed on intact skin for 8 to 48 h [91]. As we focus in this review on food allergens, we will only discuss the role of microbes in the gut and its importance for the development of atopy. Especially, the role of gut microbiota in AD is well investigated and could contribute to the understanding of the development of food allergies, as well.

In newborns, microbial colonization is dependent on maternal diet during pregnancy, type of delivery, drinking breastmilk or not, antibiotic use (pre- and postnatal) and environmental exposure, as reviewed by Perdijk and Marsland [92]. In children, colonization by E. coli in the gut at the age of 1 month was related to higher odds for the prevalence of eczema, but not for developing atopic dermatitis at 2 years. Infants colonized with Clostridium difficile had a higher risk of developing eczema, atopic dermatitis, recurrent wheeze, and atopic sensitization at 2 years of age [93]. No effect of Bifidobacteria or Lactobacilli colonization was found on the development of eczema, atopic dermatitis, wheeze, or sensitization [93]. Other studies showed that allergic children had lower prevalence of fecal Bifidobacteria [94,95,96], Lactobacilli [94,97], and a higher prevalence of *S. aureus* [94,98] and Clostridium [97,99] compared to non-allergic children. For atopic dermatitis, some studies found a decrease in Bifidobacteria [100,101], but no difference in the microbiota was found between AD patients with and without food-specific IgE [102] or in AD patients with matched controls [103,104,105]. In contrast, some studies link a reduced microbial biodiversity to the development of AD [106,107] or to atopy in general [104], although no significant effect was found for AD in this study. To induce changes in the microbiota, intervention trials have been performed with different strains of probiotics that reduced the development of atopic dermatitis [108,109,110,111,112,113,114,115] or reduced sensitization towards egg white [116], although some studies found no effect [117].

Commensal bacteria are important regulators for mucosal immunity by influencing epithelial barrier function, decreasing TSLP-production in skin via induction of Tregs in the skin and maintain homeostasis between effector and regulatory T cells in the skin, as reviewed by Salem et al. [88]. Lactobacillus casei administration was found to affect differentiation from CD8+ T cells into skin effector cells, decreased homing of these T cells to skin upon stimulation in mice, and increased the number of Tregs in the skin [118]. Oyoshi et al. found that an allergic reaction of the skin was caused by CD4+ T cells of orally sensitized mice that expressed a gut-homing profile (α4β7+) and in the draining lymph nodes switched to a skin homing profile (CCR4+) upon cutaneous exposure by OVA [119]. In addition, in children with a peanut allergy, peanut-specific T cells with a skin homing capacity showed higher proliferation compared to gut-homing peanut-specific T cells, indicating that sensitization had taken place in the skin [120]. Reducing migration capabilities of effector T cells to the skin by microbiota, while increasing Tregs in the skin, is important in preventing the development of allergic reactions in the skin.

Another way in which commensal bacteria can have an effect on allergy is by the production of short chain fatty acids (SCFAs). Acetate, propionate and butyrate are SCFAs produced by bacteria in the colon upon fermentation of non-digestible fibers. These SCFA regulate mucosal barrier function and can regulate immune responses both in the gut, as well as in the lung and skin [121,122,123,124,125]. Mice fed a high fiber diet have an increase in circulating SCFAs and showed reduced allergic inflammation in the airways [126]. In a birth cohort study, children with the highest levels of butyrate and propionate at one year of age had lower sensitization to allergens at six years of age [127]. In a recent study, human peripheral blood mononuclear cell –derived mast cells were incubated with different SCFAs in vitro [128]. Propionate and butyrate, but not acetate, were able to inhibit both IgE-mediated and non-IgE-mediated mast cell degranulation in a concentration dependent manner [128]. Furthermore, AD patients had lower SCFA production compared to control patients and several studies found that SCFA have antimicrobial effect and in particular propionate has an antimicrobial effect on *S. aureus* in vitro, as reviewed by Salem et al. [88].

So, there is no conclusive evidence that specific microbial species are responsible for the development of allergy or atopic dermatitis; nevertheless, there seems to be a crosstalk between the gut microbiota, its metabolites and the skin.

Key messages:

- There is no conclusive evidence that specific microbial species are responsible for the development of allergy or atopic dermatitis.
- Short chain fatty acids produced by intestinal microbiota are linked to reduced allergic inflammation.

## 9. Future Human Research Priorities

In this review a few important mechanisms are described that can play a role in the sensitization to food and food allergy, which are proven in murine models but not yet in humans. For the MMC9 cells there is circumstantial evidence that they are present in humans [74]. However, no flow cytometric analysis or immunohistochemistry has been performed on duodenal biopsies of food allergic patients to confirm the existence of MMC9 cells in humans.

In mice it was proven that only a cocktail of the three monoclonal antibodies against IL-25, IL-33, and TSLP can inhibit the development of food allergy in mice [72]. To our knowledge, this approach has not been tested in humans yet, although it could be very beneficial for AD patients in general, as well.

The role of environmental allergens, such as exposure to detergents as SDS, followed by HDM allergens or *Alternaria alternate*, is investigated in mice. In AD patients, exposure to HDM increased TSLP release in the skin [29], but no combination was made with detergents or other environmental allergens. Detergents are tested in vitro in human epidermal keratinocytes, and these resulted in decreased tight junction formation and barrier function of epidermal keratinocytes [129]. Therefore, it would be very interesting to investigate the effect of exposure to a combination of environmental factors, such as detergents and HDM in healthy volunteers and AD patients in a double-blind placebo-controlled study. In this set-up, skin barrier function can be addressed and atopy development could be followed as outcome. Next to this, large cohort-studies should be carried out to investigate which of the factors: skin barrier function, carriage of *S. aureus*, and having AD, is responsible for the increased sensitivity to food allergens [21]. Consequently, treatment of the skin can be optimized and sensitization to food allergens via the skin can be prevented. If these studies are performed in very young children the effect of oral ingestion prior to skin exposure of allergens should be included.

## 10. Conclusions

In this review, we highlighted the role of the skin in the development of IgE-mediated food allergy. Furthermore, we summarized the cellular and molecular mechanisms in the skin-to-gut crosstalk in the development of IgE-mediated food allergy. The site where food antigens are firstly taken up, either the skin or the gut, may cause sensitization (skin) or tolerance (gut) against this food antigen. However, sensitization towards food antigens can potentially also take place in the intestine as the result of an increased intestinal permeability. Prevention of scratching the skin is an important therapeutic target to prevent impaired skin barrier. Evidence in mouse models and clinical studies suggest that, if the skin barrier can be improved and/or the inflammation of AD can be proactively prevented, in combination with early introduction of food antigens, then the incidence of food allergy and possibly other forms of allergic diseases might be decreased.

## Glossary of Terms

ATP	Adenosine triphosphate
AD	atopic dermatitis
DC	dendritic cell
FLG	Filaggrin
HDM	House dust mite
Ig	Immune globuline
IL	interleukin
ILC	innate lymphoid cells
LPS	lipopolysaccharide
MC	mast cells
MMC9	IL-9-producing mucosal mast cell
OVA	ovalbumin
SCFA	short chain fatty acids
SDS	sodium dodecyl sulfate
*S. aureus*	*Staphylococcus aureus*
TEWL	transepidermal water loss
Th2	T helper cell 2
TSLP	thymic stromal lymphopoietin
Treg	regulatory T cell
